# Ultrasound Sensors for Process Monitoring in Injection Moulding

**DOI:** 10.3390/s21155193

**Published:** 2021-07-31

**Authors:** Mandana Kariminejad, David Tormey, Saif Huq, Jim Morrison, Marion McAfee

**Affiliations:** 1 Centre for Precision Engineering, Materials and Manufacturing (PEM Centre), Institute of Technology Sligo, Ash Lane, F91 YW50 Sligo, Ireland; Tormey.David@itsligo.ie; 2 Centre for Mathematical Modelling and Intelligent Systems for Health and Environment (MISHE), Institute of Technology Sligo, Ash Lane, F91 YW50 Sligo, Ireland; 3 School of Computing and Digital Media, London Metropolitan University, 166-220 Holloway Rd, London N7 8DB, UK; s.huq@londonmet.ac.uk; 4 Department of Electronics and Mechanical Engineering, Letterkenny Institute of Technology, Port Rd, Gortlee, Letterkenny, F92 FC93 Donegal, Ireland; jim.morrison@lyit.ie

**Keywords:** injection moulding, ultrasound sensors, sol-gel, process monitoring, lead-based chemicals, lead-free chemicals

## Abstract

Injection moulding is an extremely important industrial process, being one of the most commonly-used plastic formation techniques. However, the industry faces many current challenges associated with demands for greater product customisation, higher precision and, most urgently, a shift towards more sustainable materials and processing. Accurate real-time sensing of the material and part properties during processing is key to achieving rapid process optimisation and set-up, reducing down-times, and reducing waste material and energy in the production of defective products. While most commercial processes rely on point measurements of pressure and temperature, ultrasound transducers represent a non-invasive and non-destructive source of rich information on the mould, the cavity and the polymer melt, and its morphology, which affect critical quality parameters such as shrinkage and warpage. In this paper the relationship between polymer properties and the propagation of ultrasonic waves is described and the application of ultrasound measurements in injection moulding is evaluated. The principles and operation of both conventional and high temperature ultrasound transducers (HTUTs) are reviewed together with their impact on improving the efficiency of the injection moulding process. The benefits and challenges associated with the recent development of sol-gel methods for HTUT fabrication are described together with a synopsis of further research and development needed to ensure a greater industrial uptake of ultrasonic sensing in injection moulding.

## 1. Introduction

Injection moulding is an exact and economical method for producing large volumes of plastic products. Injection moulded parts do not require any post-processing and it has the advantage of being a “net shape” process [1]. It has been estimated that more than one-third of the world’s plastic products are manufactured by this method [2]. Accurate real-time control of the process is essential in high-value applications, such as medical devices, which demand high precision in the part dimensions. Process monitoring and control is also increasingly important because of a need to use more sustainable raw materials, such as recycled polymers, which have variable feed properties, and bio-based materials which tend to be thermally sensitive and more difficult to process. For this purpose, various sensors have been applied for real-time and inline process monitoring in the injection moulding process such as pressure sensors, temperature sensors and ultrasonic sensors.

Commercial injection moulding processes typically incorporate temperature and pressure sensors at various points in the injection moulding machine. However, these provide limited information and may not be sufficient to enable effective control of the process to avoid common defects such as warpage and shrinkage of the moulded components [3]. Conventional thermocouples are known to provide only a surface temperature measurement, which is usually dominated by the temperature of the metal mould or barrel rather than reflecting the true bulk temperature of the polymer melt [4]. Knowledge of the bulk melt temperature is, however, important to prevent defects such as polymer degradation or incomplete filling. The cavity pressure should also be monitored to avoid part defects such as flash (overflow of polymer in the mould); however pressure measurements via conventional diaphragm pressure sensors are influenced by the layer of the frozen polymer at the cavity wall and can be lower than the exact melt pressure [4]. Moreover, these sensors are invasive, requiring holes and modifications to be cut into the mould—they are often challenging to physically fit due to the limited space available in the mould, once cooling channels and ejection pins and so on have been incorporated.

Ultrasound sensors have several advantages over conventional temperature and pressure sensors in injection moulding. They are non-invasive and they can provide not only rich information about polymer morphology [5] and the physical process parameters but also the exact temperature and pressure of the polymer melt. With regard to temperature measurement, ultrasonic sensors are not affected by heat conduction and convention as thermocouples are, nor are they affected by the absorbance and reflectance of the material as infrared temperature sensors are [6,7,8]. The exact pressure can also be measured since ultrasonic signals can propagate through the melt and are not affected by the frozen layer fraction. Hence, this review paper is explicitly focused on the various research on ultrasonic sensors used within the injection moulding process.

The injection moulding process comprises four main stages [1]: the first stage is filling, when the polymer pellets are melted and conveyed along a screw in a heated barrel. The second stage is packing, where extra polymer is injected into the mould cavity to compensate for the polymer shrinkage that occurs on solidification. The next stage is cooling, which provides time for the polymer to cool and solidify. The final stage is ejection, when the part that has been inserted in the immobile moulds is ready to be ejected by the ejector pins.

There are process control challenges associated with each stage. During the filling stage, the melting behavior of solid polymer in the screw influences the part quality. The screw consists of a feed zone that contains the plastic pellets, a melting zone where the plastic pellets change to a continuous melt, and a metering zone in which the melt should attain uniform temperature and morphology to inject into the cavity. In the melting zone, there exists a solid bed and a melt bed, and the progression of the solid bed/melt bed ratio is important in achieving a homogeneous melt without viscosity variations and without degrading the polymer. Consequently, the monitoring of the melting process in the filling stage can be important to prevent issues which affect the later stages of the process [2]. The packing stage may be either static or dynamic. Dynamic packing is a method for producing dynamic pressure in the cavity, which improves the mechanical properties of moulded products such as tensile strength [9]. Because the melt is injected into the cavity repeatedly by two hydraulic pistons, a highly-oriented polymer morphology is obtained [10,11]. Whichever method is used, the packing pressure should be tightly controlled to avoid defects such as flash and incomplete filling in the process. During the cooling stage, shrinkage occurs due to the decline in cavity pressure and a gap is formed between the cavity and the mould. Monitoring the gap formation is useful to monitor and optimize the part shrinkage.

All these stages occur quickly, and the cycle time may be less than one minute, depending on the cavity size and shape. There are many process parameters that should be monitored and controlled simultaneously including melt temperature and pressure, cooling rate, packing pressure, cavity temperature, holding time, polymer morphology, and so forth. Computer Aided Engineering (CAE) software is well developed for the process and the off-line optimization of process settings can be carried out using a combination of simulation and Design of Experiments approaches [12,13,14,15]. However, variations are inherent in polymer materials and inline monitoring and control of the process is essential for achieving high precision parts and in any process where variable feedstock (e.g., recycled material) is being processed. Hence, a real-time method that can predict the occurrence of undesired warpage and shrinkage, inline or just after part ejection is highly desirable.

**Figure 1:**
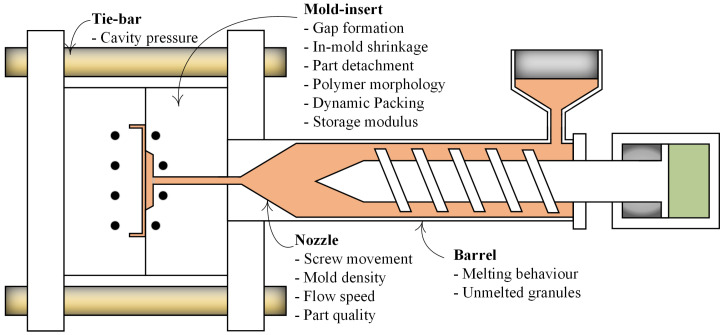
Monitored parameters with ultrasonic sensors at the different locations of injection moulding.

In the following sections, the ultrasound mechanism and the relationship between ultrasound propagation properties and different injection moulding process parameters are described. The principles and operation of both conventional and high-temperature ultrasound transducers (HTUTs) are reviewed together with their impact on improving the efficiency of the injection moulding process. The benefits and challenges associated with the recent development of sol-gel methods for HTUT fabrication are described together with a synopsis of the further research and development needed to inform greater consideration for the industrial uptake of ultrasonic sensing in injection moulding. A general overview of the application of ultrasonic probes at different injection moulding machine’s locations, such as mould insert, barrel, nozzle and tie bars, is summarised in Figure 1.

## 2. Properties of Ultrasound Wave Propagation

Ultrasound waves are high-frequency mechanical waves in the region above 20 kHz [16]. Ultrasound transducers are based on the converse piezoelectric effect, whereby a voltage pulse applied to the surface of a piezoelectric material results in a mechanical displacement in the material for the duration of the pulse. Thus, in response to a train of pulses at the required frequency, an ultrasound wave is generated [17]. The most common types of ultrasound waves that propagate through materials are: Longitudinal waves, Shear waves, Rayleigh or Surface Acoustic Waves, and Lamb waves [18]. Rayleigh and Lamb waves travel near the surface of solids and are unsuitable for the bulk analysis of polymer melts. Longitudinal waves are preferred to shear waves in polymer melts since shear waves attenuate too quickly in polymers, giving little penetration into the media [19].

The Ultrasound wave is characterised by two properties: attenuation and velocity. The attenuation of ultrasound can be defined by the decay in the amplitude of the ultrasound signal as it travels through a medium. Attenuation can be due to energy dissipated by conversion to heat, absorption, and scattering of ultrasound waves [19]. The velocity can be measured by time of flight, which is the travel time of an ultrasound signal through a medium. The velocity and attenuation of an ultrasound wave are sensitive to the properties of the media through which it propagates, and several works have explored the relationships between the propagation properties of ultrasound signals to properties of polymer media with the aim of exploiting ultrasound transducers for rapid and non-destructive polymer characterisation. In 1964, Thurston [20] proposed an equation for ultrasound velocity $V_{L}$ of longitudinal wave based on bulk moduli (*K*), shear moduli (*G*), and the density (ρ) of a polymer media:

(1) $$V_{L}=\frac{1}{\rho }{\left(K+\frac{4G}{3}\right)}^{1⁄2}.$$

Since the shear moduli in polymers is negligible, the sound velocity can be expressed as:

(2) $$V_{L}={\left[\frac{k}{\rho }\right]}^{1/2}={\left[\frac{1}{\rho k}\right]}^{1/2}.$$

*k* is the adiabatic compressibility, which can be calculated as [21]:

(3) $$k=\left(\frac{1}{v}\right)\left[{\left(\frac{\partial v}{\partial P}\right)}_{T}+\left(\frac{T}{c_{p}}\right){{\left(\frac{\partial v}{\partial T}\right)}_{p}}^{2}\right],$$

where *v* is the specific volume and $c_{P}$ is the specific heat capacity at constant pressure *P*. The specific volume, v(T,P) at temperature *T* and pressure *P* can be obtained from the Tait equation [22], $v_{0}\left(T\right)$ is zero-pressure isotherms, B(T) is a function of temperature, which is independent of pressure and C is a universal constant:

(4) $$v\left(T,P\right)=v_{0}\left(T\right)\left[1-C\ln\left(1+\frac{P}{B(T)}\right)\right]+v_{t}\left(T,P\right).$$

**Figure 2:**
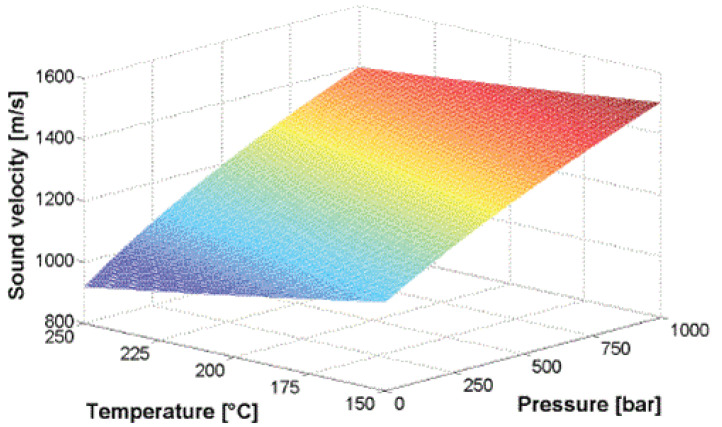
Sound velocity based on pressure and temperature in PP [22].

Hence, by combining (2)–(4), the longitudinal velocity of ultrasound in the polymer can be derived as a function of pressure and temperature. Praher et al. in 2014 [22] simulated the contour of sound velocity based on temperature and pressure for polypropylene, presented in Figure 2. It should be noted that extracting temperature and pressure information from ultrasound echoes is an indirect measurement, which requires a model specific to the material being investigated.

Moreover, in 1964, McSkimin proposed equations for longitudinal and shear waves, related to material density (ρ), wavelength (λ), attenuation (α) and velocity (*c*) of ultrasound signals as follows, where ($V_{L}$) is the longitudinal velocity and ($V_{S}$) is the shear velocity [23]:

(5) $$V_{S}\;=\frac{\rho c^{2}\left[1-{\left(\frac{a\lambda }{2\pi }\right)}^{2}\right]}{{\left[1+{\left(\frac{a\lambda }{2\pi }\right)}^{2}\right]}^{2}}$$

(6) $$V_{L}=\frac{2\rho c^{2}\left(\frac{a\lambda }{2\pi }\right)}{{\left[1+{\left(\frac{a\lambda }{2\pi }\right)}^{2}\right]}^{2}}.$$

The reflection (*R*) and transmission (*T*) coefficients of an ultrasound signal between the interface of two media by using values of acoustic impedance can be calculated as: [24]:

(7) $$R=\frac{z_{1}-z_{2}}{z_{1}+z_{2}}$$

(8) T=1−R.

$z_{i}$ is the ultrasound impedance of the *i*th medium, which can be defined based on density $\rho_{i}$ and wave velocity $V_{i}$:

(9) $$z_{i}=\rho_{i}V_{i}.$$

The reflection coefficient indicates the ultrasonic wave reflected back through two media, while the transmission coefficient is the wave transmitted through the interface. The velocity of a longitudinal ultrasound wave in a solid, related to Young’s modulus (E), Poisson’s ratio (σ), and material density (ρ) can be defined as [18]:

(10) $$V_{L}=\sqrt{\frac{E(1-\sigma )}{\rho (1+\sigma )(1-\sigma )}}.$$

**Figure 3:**
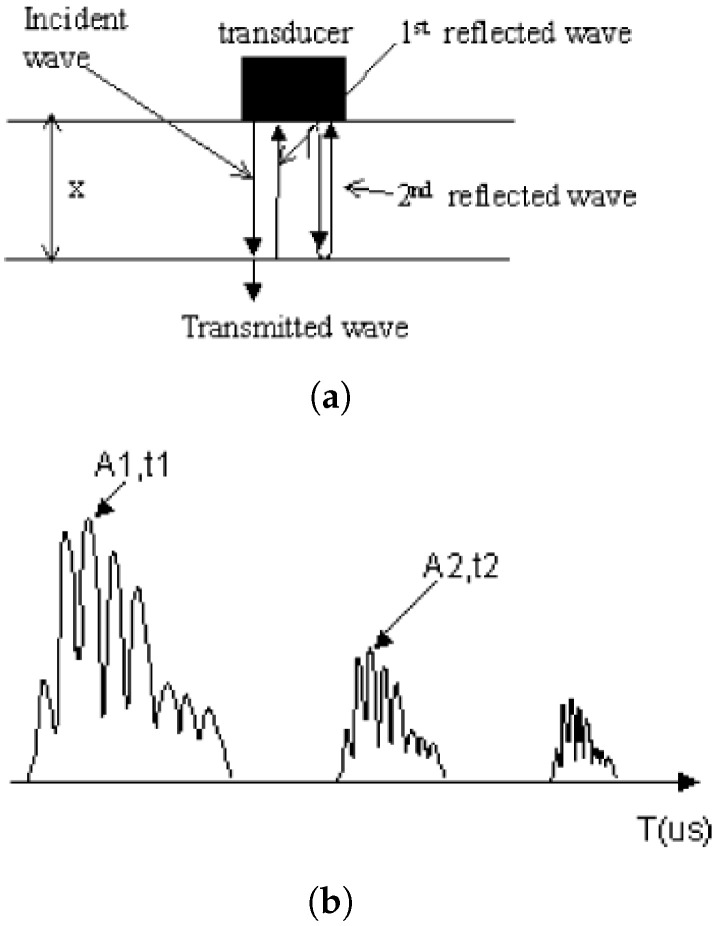
(**a**) Ultrasound propagation; (**b**) Ultrasound echo signals [18].

The attenuation of the ultrasound signal (α) and its velocity (V) can also be calculated by the ratio of the amplitudes of two successive attenuating echoes through melt as shown in Figure 3a, *X* is the thickness of the polymer substrate, $A_{1}$ and $A_{2}$ are the amplitudes of the signals, $t_{1}$ and $t_{2}$ are the corresponding transit times as shown in Figure 3b [18]:

(11) $$V=\frac{2X}{t_{2}-t_{1}}$$

(12) $$\alpha =\frac{20{log}_{10}(A_{1}/A_{2})}{2X}.$$

Hsu, in 1974, suggested that using ultrasound shear waves has some advantages for measuring stress in solids [25]. Zhang in 2019 used this theory and the equation derived by Sayers [26] to estimate the stress (σ) of the tie bar in the injection moulding process based on the propagating speed of the ultrasonic wave [27]:

(13) $$\rho_{0}.V_{\sigma }^{2}=\lambda +2\mu +\frac{\sigma }{\left(3\lambda +2\mu \right)}\left[2l+\lambda +\left(\lambda +\mu \right)\left(4m+4\lambda +10\mu \right)\right],$$

where $\rho_{0}$ is the density of the tie bar, and $V_{\sigma }$ is the ultrasound velocity when the tie-bar tolerates a stress of σ. The remaining parameters are material constants in which μ and λ are the Lamé constants of the material (material-dependent constants arising in strain-stress relationships) and *l*, *m*, and *n* are the Murnaghan constants of the material (constants related to elastic deformation).

A summary of the measured ultrasonic properties and the corresponding related material properties, along with the relating equation, is presented in Table 1.

## 3. Conventional Ultrasonic Transducer (UT) Applications in the Injection Moulding Process

The application of conventional ultrasonic sensors in injection moulding has been investigated since 1997. These sensors have been applied at different locations of the injection moulding machine including the mould insert, barrel, tie bars and nozzle for the inline monitoring of the process parameters, polymer morphology, and stages of the process.

### 3.1. Application of Conventional Ultrasonic Probes at the Mould Insert and Barrel

The first application of conventional ultrasonic sensors in injection moulding was to monitor the gap formation between the cavity wall and the part [24]. Here, UTs were installed on the mould and the core. The gap was monitored by the change in the ultrasonic reflection and transmission coefficients. The contact time between the cavity wall and polymer based on defined good-contact and partial-contact was also extracted by measuring the signal amplitude. The authors verified that the ultrasonic sensors had better performance for detecting gap development than conventional pressure sensors. A cavity pressure sensor can only detect the melt flow arrival by the surge in pressure, during gap formation the pressure drops to zero, so the pressure sensor is not capable of monitoring the development of the gap.

Brown et al. [28] modified an ultrasonic sensor with a buffer rod to make it suitable for mounting in the nozzle of an injection moulding machine. Comparing the result with thermocouples and infrared sensors, it was concluded that the ultrasound sensors provide more information about the melt since they propagate through the melt in the cavity. For example, it was shown that the true melt temperature in the nozzle is higher than that measured by the infrared sensor.

**Figure 4:**
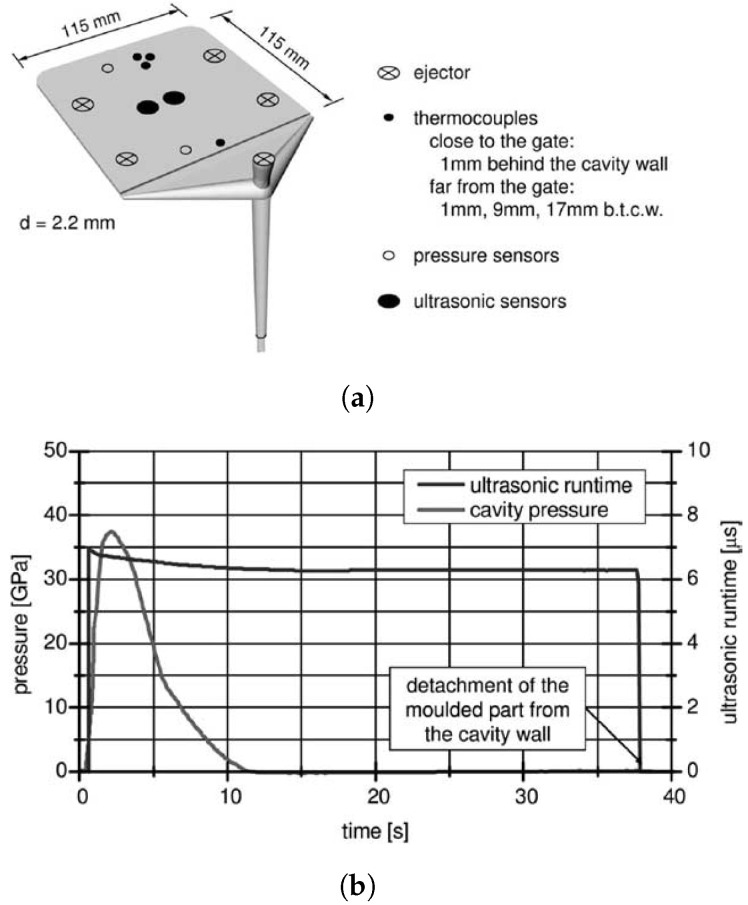
(**a**) Location of the thermocouples, pressure sensors, and ultrasonic sensors at the moulded part; (**b**) Comparison of Ultrasound echoes and pressure sensor [29].

Michaeli et al. [29] also compared the performance of conventional pressure and temperature sensors to ultrasonic sensors. The moulded part and the position of the sensors are shown in Figure 4a. Three different types of polymeric materials were investigated: amorphous (ABS, PPMA, PC), crystalline (PP, PA6), and fiber-reinforced crystalline (PA66GF30). In the first part of the experiment, the cooling time was calculated by a cooling equation from [30] for each material, and the holding pressure was selected such that the part detached from the ultrasonic probe location at the end of calculated cooling time.

In the second part of the experiment, the influence of process parameters was investigated by varying the cavity wall temperature and injection speed at two levels and holding pressure at three levels. The first experiment illustrated that the ultrasonic signals could provide more information on solidification and part detachment than pressure sensors, since the echoes can propagate through the melt and the time of part detachment from the wall can be accurately determined (Figure 4b). Amorphous and crystalline materials could be distinguished by the comparison of the ultrasonic amplitude during the different solidification processes for each material. It was observed that the ultrasonic amplitude had a local minimum for the amorphous materials in the holding pressure phase, while the amplitude dropped continuously until the crystallization phase and part detachment of the crystalline material. The second experiment, where two different levels of cavity wall temperature were investigated, indicated that as the temperature decreases, the ultrasonic velocity increases significantly. Secondly, the variation in injection speed did not significantly affect the ultrasonic velocity or amplitude. Finally, the variation in holding pressure is the only parameter that directly influences the time of part detachment from the mould wall while not affecting the ultrasonic velocity substantially.

He et al. [31] conducted a similar experiment to investigate the effect of injection pressure and temperature on the solidification of three types of polymer materials: crystalline (Linear low-density polyethylene LLDPE), non-crystalline (Polymethacrylate PMMA), and a polymer blend (Isotactic polypropylene/ethylene–octane copolymer iPP/POE). An ultrasonic study of dynamic-packing injection moulding was also carried out. The stages of the injection moulding process were monitored for the different materials by studying ultrasound amplitude and attenuation. In static packing injection moulding of PMMA, a decline in ultrasonic attenuation and an increase in the amplitude was observed after the packing stage. This was attributed to a gradual solidification of the polymer chains resulting in an increase in elasticity. Conversely, for LLDPE, an increase in attenuation and a reduction in amplitude was observed, since crystallites start to form on cooling, and as the number of crystallites increase the attenuation is enhanced. The effect of mould temperature on solidification was also investigated. The results showed that at a lower mould temperature, and hence a higher cooling rate, the faster formation of crystallites in LLDPE could be observed by the more rapid attenuation in ultrasonic velocity. The phase morphology of the iPE and POE polymer blend was also observed by the ultrasonic attenuation. The dispersed phase particles are enhanced during solidification, which means an increase in scattering loss and hence an increase in ultrasonic attenuation. During the dynamic packing process, the fluctuation of cavity pressure is related to ultrasonic velocity and amplitude. An increase in solidification time could be observed in a slow rise in the ultrasonic velocity relative to the solidification phase in the static packing process. In the dynamic packing process, melt is injected to the cavity repeatedly, which induces more heat and hence prolongs the cooling and solidification.

Real-time monitoring of the melting behaviour in the barrel of an injection moulding machine was also investigated by Altman et al. [32]. First, they proposed a numerical simulation of the melting process of a polyamide 6 (PA6) material based on the melting model of Tadmor and Gogos to model the solid/melt bed ratio in the barrel [33]. The solid bed and melt pool were monitored in a process with three ultrasonic probes and seven pressure sensors along the barrel. A comparison of the results from the sensors and simulation indicated good agreement between the experimental data and the simulation.

Recently, in 2021, Zhao et al. [34] used ultrasound sensors to measure the melt temperature inline. A new approach was proposed by the correlation of ultrasound principles and the PVT equation in (4). They used thermocouples and an infra-red temperature sensor to compare the melt temperature measurement with the ultrasound method. Under the same process parameters, the thermocouple failed to measure the temperature accurately, and the results from the infrared sensor and the proposed method illustrated a good agreement with a 5.5% error. The effect of different process settings was also investigated and indicated an error below 7.5%, which was attributed to the different locations of the sensors. For investigation of the influence of different types of material on the accuracy of melt temperature measurement, a crystalline LDPE and an amorphous PVG were compared. Here, the error was higher for the PVG due to the difference in the material properties. A study has also investigated the further application of ultrasound sensors to measure the level of plastic pellets in the storage tank of an injection moulding machine [35]. The results showed a high accuracy of the sensors above 95% for measuring the level of the pellets.

### 3.2. Application of Conventional Ultrasonic Probes at the Barrel, Nozzle, and Tie Bars

**Figure 5:**
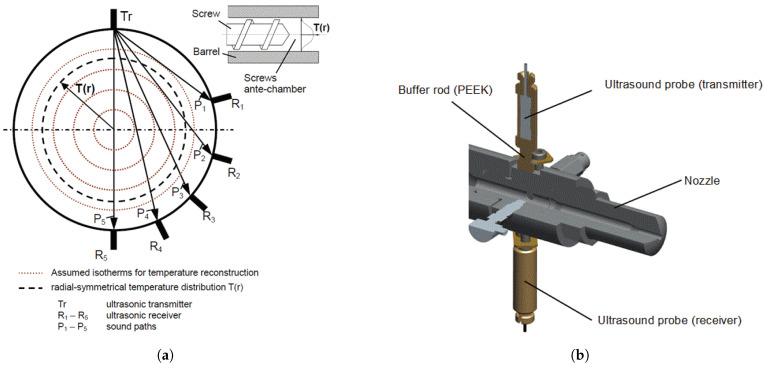
(**a**) Fan-shaped array of the ultrasonic probe at the barrel, and (**b**) ultrasonic probes between the nozzle and the barrel [22].

The application of ultrasonic probes in monitoring the melting and conveying processes in the screw of the injection moulding process has also been investigated. Praher et al. [22] proposed a fan-shaped ultrasonic transmitter and receiver array along the barrel for measuring the two-dimensional temperature in the screw antechamber, shown in Figure 5a. The temperature of each ring segment in Figure 5a was calculated through numerical simulation using the fact that the transit time of ultrasound echoes in each ring segment is related to the temperature and pressure. For the detection of unmelted granules at the screw, two ultrasonic probes, with buffers for tolerating high temperature, were inserted between the nozzle and the barrel (Figure 5b), and the variation in the ultrasonic attenuation was correlated to the number of unmelted granules. The solid bed and melt bed ratio can also be observed from ultrasonic reflection signals; the continuous signal from the experiment clarified the absence of the solid bed.

Ultrasonic probes have also been used for measuring the clamping force and cavity pressure in tie bar injection moulding. Zhao et al. [36] proposed a mathematical model for measuring the clamping force inline based on ultrasound principles and (13). For this purpose, ultrasonic probes were glued to the four tie bars of the injection moulding machine and the measured ultrasonic velocity was used to calculate the stress in the tie bars. Zhang et al. [27] further developed this method to measure the cavity pressure. The cavity pressure was derived from the stress in the tie bar by the fact that the melt pressure and clamping force are transferred to the tie bars by the mould and platen of the machine. Comparison with a Kistler cavity pressure sensor indicated a measurement error of 4.3%.

Layer thickness in a water-assisted co-injection moulding process, which is a process for the production of hollow components with multi-layered walls, has also been investigated by ultrasound sensors [37]. The authors proposed a model for ultrasound propagation in a layered polymer from a co-injection process. Using this model, they calculated the amplitude of the ultrasound signal reflected from successive interfaces by defining a transfer function for the medium in each layer. For the evaluation of the calculated signal obtained from the transfer function, the normalized root mean square has been used as an objective function to compare the measured signals from the probe and the calculated signal. Two objective functions were employed to narrow the search space continuously and by finding the solution, the optimum parameter for calculation of layer thickness has been found.

A recent study has also indicated the potential application of ultrasonic signals in a microcellular injection moulding process [38], which is a promising process for producing lightweight, foamed products containing numerous bubbles or ‘cells’ on the micron-scale. The ultrasonic probe was positioned on the outside surface of the mould and the ultrasonic signals were used for inline characterization of cell size, surface roughness, and skin layer thickness which are critical parameters affecting the dimensional stability, aesthetics and mechanical properties of parts produced by this advanced injection moulding process.The ultrasonic signals were shown to be a highly repeatable, rich and sensitive source of information on the foaming process with the advantages of being low-cost, non-destructive and facilitating real-time characterisation of the product.

The process and material parameters, which can be monitored by conventional UTs, are summarized in Table 2, which highlights the range of useful information that can be deduced from ultrasonic probes in different locations of the injection moulding machine.

## 4. High-Temperature Ultrasonic Transducers (HTUT) by Sol-Gel Technique

Conventional ultrasound transducers are most commonly made from a piezoceramic material such as Lead-Zirconate-Titanate (PZT), Zinc Oxide (ZnO), Barium Titanate (BaTiO${}_{3}$) and Lead Titanate (PbTiO${}_{3}$) [39,40,41]. A thin-film coating can be fabricated by thermal or plasma spraying, electrochemical etching or physical vapour deposition methods such as sputtering [42,43,44,45]. These methods can only be applied on flat structures and, in the case of plasma spraying, further post-deposition is required since the produced films are porous. Moreover, the thin-film ultrasonic sensors fabricated via these methods operate in a limited frequency range, are not suitable for high temperature areas, and have limited sensitivity and piezoelectric properties [40,45]. Sol-gel is an alternative fabrication method which results in improved properties for ultrasound transducers. In the sol-gel method, films are formed from mixed solutions in a suitable solvent and a hydrolysis reaction is used to produce a gel. Additives are employed to control the viscosity and surface tension. The gel can be coated on a desired substrate by different methods such as spinning, dip coating and spray coating. Thermal treatment is also required to develop the structure [46].

The conventional sol-gel method can be used for the fabrication of thin-films up to 0.5 µm in a single layer; however, it is not possible to produce a thicker film without cracks. In 1997, Barrow et al. [47] invented a method for the fabrication of a crack-free thick film (thicker than 10 µm). The proposed method was to disperse a ceramic powder into the sol-gel solution. Barrow’s invention was an important step forward in achieving a more economical and versatile method for a thick ceramic coating without cracks, resulting in improved properties for ultrasonic transduction. Relative to conventional ultrasonic probes, transducers fabricated via the sol-gel technique are miniature; they can be used on curved and flat shapes; they have higher sensitivity, higher energy densities [48], a high signal-to-noise ratio (SNR) and a high thermal tolerance. Another advantage of this fabrication method is that no additional couplant is required as the ceramic powder makes a strong bond between the sol-gel film and the substrate, resulting in a crack-free film with good acoustic coupling [46,49].

The fabrication of an ultrasonic sensor with the sol-gel technique can be divided into the following stages:

1. The solution should be prepared, and the desired ceramic powder should be added to the solution and dispersed by a stirrer;
2. The film should be deposited on the desired substrate by a coating process, and thermal treatment should be also applied;
3. Repeated coating layers should be applied to reach the desired thickness;
4. The film’s stability and properties should be characterised and the top electrode should be placed for electrical connection;
5. The film should be electrically poled. Methods include DC Corona poling, high temperature corona poling and DC power.

This fabrication technique can be applied at high temperatures and on curved areas of the injection moulding process such as the wall of the injection barrel. This is highly desirable since obtaining the exact melt temperature can help prevent polymer degradation and incomplete filling in this stage of the process.

### 4.1. Application of Sol-Gel Ultrasound Sensors in the Injection Moulding Process

As mentioned above, the benefits of using these sensors in the injection moulding process is that, compared to conventional probes, they are miniature, can tolerate high temperature, can be applied on curved shapes, and can thus be applied in different areas of the machine where conventional probes are difficult to fit. In the following sections, the application of sol-gel fabricated ultrasonic sensors on the different locations of the injection moulding machine, such as barrel, mould inserts and nozzle, will be discussed.

#### 4.1.1. Application of Sol-Gel Sensors on the Barrel and in Mould Inserts

**Figure 6:**
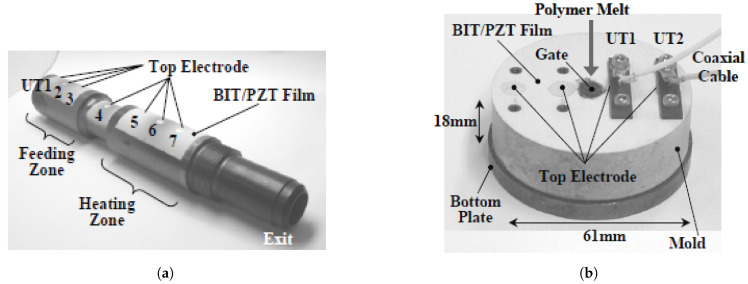
Fabricated sol-gel ultrasound sensors in the micromoulding process (**a**) on the barrel and (**b**) at the mould insert [50].

The first use of sol-gel ultrasonic transducers in the injection moulding process was in a micromoulding machine for creating parts with micrometer dimensions by Kobayashi et al. [50]. They fabricated seven ultrasonic transducers on the barrel; six sensors were located on the feeding and heating zones, and they also placed a sensor between them (Figure 6a). Two sensors were also fabricated on the mould insert of the machine, as shown in Figure 6b. They successfully measured the ultrasonic signals at the barrel and the velocity of the polymer melt in the cavity during solidification for the polyethylene moulded part.

In 2005, Whiteside et al. [51] conducted a similar experiment on a micromoulding machine. They fabricated sensors on the barrel and in a mould insert to monitor polymer degradation and filling incompleteness by monitoring the variations of ultrasonic amplitude and velocity over the process time. The in-process degradation of POM (Polyoxymethylene) was deliberately induced by setting the melt temperature higher than recommended. The ultrasonic velocity in the mould insert and in the barrel was monitored during the process. The ultrasonic velocity measured in the mould insert increased suddenly after 6 min, which was correlated to a gradual increase in part thickness due to the degradation of the polymer.

**Figure 7:**
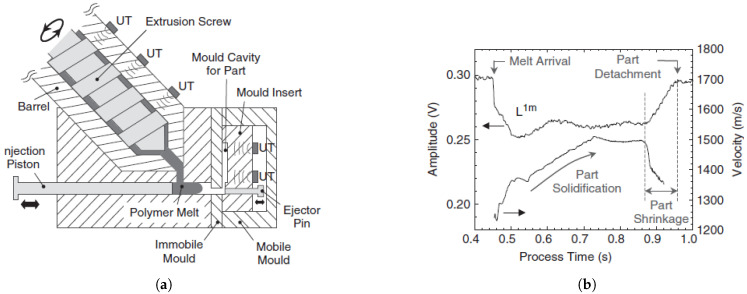
(**a**) Fabricated sol-gel UTs at the nozzle and Mould insert; (**b**) The velocity and amplitude of ultrasonic sensor vs. Process time [52].

Ono et al. [4] also investigated this type of sensor’s performance for a simple POM rectangular part in a micromoulding process. They used seven sensors fabricated by a sol-gel technique on the barrel and two sensors on the mould insert of a mobile mould. Figure 7a shows the sensors fabricated on the injection moulding machine. The nth round trip longitudinal ultrasonic wave echoes reflected from the cavity surface were called $L^{\mathrm{nm}}$, and the ones propagating in the polymer and reflected from the immobile insert were called $L^{2\mathrm{nm}}$. Figure 7b shows the ultrasound velocity versus process time for the first ultrasound transducer at the mould insert and the amplitude variation of the first reflected echo from the mould insert $\left(L^{1m}\right)$. In less than half a second, the polymer arrived in the cavity, and a portion of the ultrasound signal propagates through the polymer and reaches the immobile mould. The solidification of the part could be monitored by an increase in ultrasound velocity up to 1500 m/s during the cooling stage. After solidification, the amplitude of $L^{1m}$ gradually rises, showing the part detaching from the mould wall because of shrinkage. Finally, a reduction in velocity suggested shrinkage since an air gap developed between the cavity and the part.

In 2006, Kobayashi used ultrasound transducers fabricated by a sol-gel technique for the first time in a conventional (not micromoulding) injection moulding process [53]. He used Bismuth Titanate/Lead Zirconate Titanate (BIT/PZT) film and embedded four sensors in a mould insert of an injection moulding machine. The sensors successfully monitored the flow front arrival, the velocity of the flow front, the mould opening time, detachment of the part from the mould walls, and various stages of injection moulding by variation in the amplitude of the reflected signal from the HTUT.

Zhao et al. [54] fabricated a novel high-temperature ultrasonic transducer called the $L-S_{⊥}-S_{\|}$ probe, capable of measuring longitudinal and shear waves simultaneously. They inserted two ultrasound probes measuring only longitudinal waves (‘L’ probes) and two $L-S_{⊥}-S_{\|}$ probes in the mould insert of an injection moulding machine producing a rectangular High-density Polyethylene (HDPE) moulded part. The ultrasonic shear wave velocity for vertical $\left(V_{V}\right)$ and horizontal $\left(V_{H}\right)$ flow directions based on the shear stiffness $(G_{xy}$ and $G_{zy})$, and material density (ρ) can be expressed as:

(14) $$V_{H}=\sqrt{\frac{G_{xy}}{\rho }}$$

(15) $$V_{V}=\sqrt{\frac{G_{zy}}{\rho }}.$$

The experiment was conducted over different injection moulding conditions. Besides the observation of different stages of injection moulding, the other experimental findings can be summarized as follows:

- The longitudinal velocity increases under higher temperatures and higher injection speeds, because of the change of HDPE morphology with different process settings.
- A considerable difference in echo time was associated with a higher storage modulus since the slow velocity along the flow directions indicates weak mechanical properties and high velocity along the flow direction indicates strong mechanical properties based on the (14) and (15).
- As the injection speed increased, the longitudinal and shear ultrasonic velocities differed significantly.
- Comparison of the time delay in parallel and perpendicular directions to the melt flow indicated a higher storage modulus in the perpendicular direction. This was attributed to the formation of crystalline lamellae in the perpendicular direction, which was confirmed by Scanning Electron Microscopy.

Hence, ultrasound monitoring was shown to be useful for monitoring polymer morphology and the resulting mechanical properties.

#### 4.1.2. Application Sol-Gel Ultrasonic Sensors at the Nozzle

The high-temperature and non-destructive features of HTUTs facilitated their application at the nozzle of the injection moulding process, which is not feasible with conventional ultrasonic sensors because of the limited space, high pressure, and temperature. Wu et al. [55] fabricated a pair of HTUT sol-gel films in the nozzle side of an injection moulding process to monitor the dynamic characteristics of the process at the nozzle, including the dynamic flow speed and the static density of the polymer melt in real-time.

**Figure 8:**
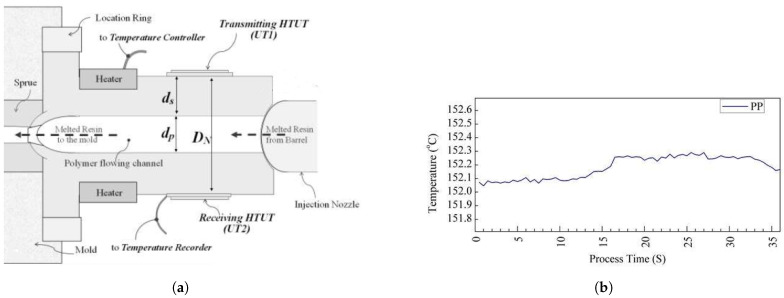
Fabricated sol-gel ultrasound sensor at the nozzle; (**a**) cross-section of the nozzle with the probes; (**b**) the measured temperature for PP by a thermocouple [56].

In 2017 [56], they modified the extension nozzle of an injection moulding machine to a T-shaped nozzle which provides a space for installing sensors. Figure 8a indicates the designed extension nozzle with the ultrasonic transducer and the signal echoes for a polypropylene (PP) moulded part. They used the sol-gel spray technique to fabricate an ultrasonic film at the nozzle, capable of tolerating temperatures up to 350 °C and pressure up to 300 bar.

**Figure 9:**
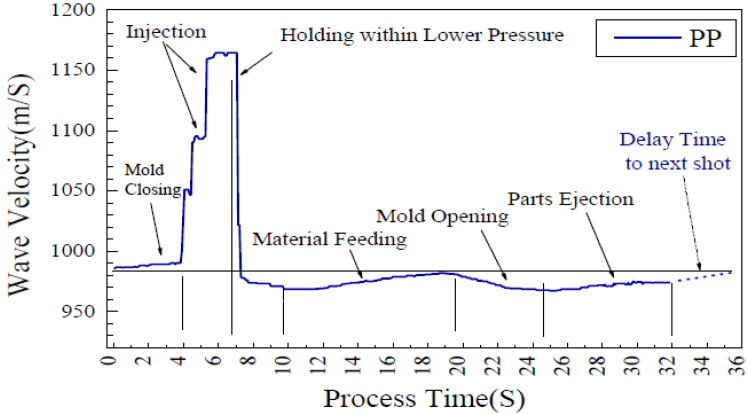
Velocity of ultrasonic signal at the nozzle during injection moulding process [56].

They investigated the ultrasound echoes reflected and transmitted through the extension nozzle, molten polymer, and air interfaces. A thermocouple was also installed at the surface of the extension nozzle for comparison. The result showed that the thermocouple lacked sufficient sensitivity for effective monitoring of the process. As shown in Figure 8b, where the set temperature for polypropylene at the extension nozzle was about 225 °C, the thermocouple measured a temperature of around 152 °C. In contrast, the ultrasonic sensors at the nozzle were capable of monitoring different stages of injection moulding (Figure 9) including the mould close time, injection, packing, solidification and ejection stages, screw movements and the mould open time. These different stages could be observed by the variation of velocity and amplitude in the ultrasonic signal over the process time.

**Figure 10:**
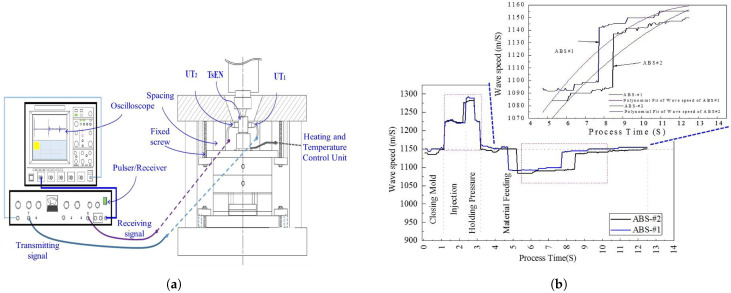
Fabricated sol-gel ultrasound sensor at the extension nozzle; (**a**) the T-shaped extension nozzle and data acquisition system for ultrasound sensor; (**b**) ultrasonic velocity vs. process time for ABS#1 & ABS#2 with different injection speed [52].

Recently, in 2020, Cheng et al. [52] further developed the research on the application of a T-shaped extension nozzle by investigating the effect of material type and various process settings on the part quality. Two HTUTs were fabricated on the extension nozzle and different injection speeds and heating temperatures were investigated for Polypropylene (PP) and ABS. Figure 10a shows the experimental set-up for measuring the ultrasound velocity. Figure 10b shows the ultrasound velocity versus process time plot for the ABS material with different injection speeds. For ABS#1, the injection speed is 45/65 m/s, and for ABS#2 the speed is 45/85 m/s. At the injection stage from 1.1 s to 2.3 s, the same ultrasound velocity is observed for both conditions because of the high viscosity of ABS. In the packing stage from 2.3 to 3.2 s, the ultrasonic velocity increased significantly. The ultrasound velocity decreased at 3.2 s due to the retraction of the screw. Finally, from 3.3 to 12.5 s, a low ultrasonic velocity can be observed during the feeding stage for both ABS#1 and ABS#2.

**Figure 11:**
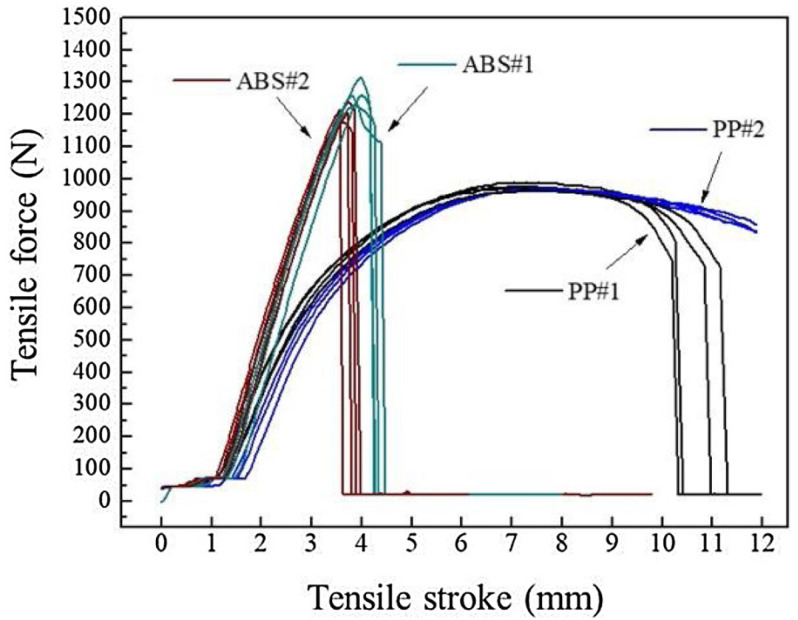
Tensile test for PP and ABS moulded parts with different injection speed [52].

A polynomial fit was applied to the ultrasound velocity against time for both types of ABS in the feeding stage and is illustrated in Figure 10b to underscore the variation of velocity for ABS#1 and ABS#2. The same experiment for PP showed that the ultrasonic velocity in ABS was higher than in PP because of different compressibility and density, and secondly the variation in ultrasonic velocity over the process at different injection speeds in the feeding stage was higher in PP than in ABS. A tensile test was also applied to investigate the relationship between the process parameters, ultrasound signal, and the part quality in the feeding stage. The tensile test result showed that for PP, a ductile material, the components produced at higher injection speed did not break as they did for the components produced at the lower injection speed. However, for the ABS, a brittle material, the components produced at greater injection speed had a higher deflection distance (Figure 11). The variation of pull strength as the injection speed changed was greater for ABS than for PP, which correlated to differences in the ultrasound signals at the feeding stage for ABS and PP. The results suggest that the changes in ultrasonic velocity observed at different injection speeds are a good indicator of the resulting tensile properties of the components.

Table 2 summarises the process and material parameters which can be deduced by both conventional and sol-gel fabricated UTs.

**Table 2:** Process and product information extracted from different types of ultrasonic probes in different locations of the Injection moulding.

Sensor’s Type	Sensor Location	Investigated Process/Material Parameters	Reference
Conventional Ultrasonic Probe		1. Gap formation	[24]
		2. Contact time	[24]
		3. Timing of different process stages	[24,29,31]
	Mould	4. Part detachment	[29]
		5. Solidification time in dynamic and static packing	[29]
		6. Melt Temperature	[34]
		7. Detection of crystalline & amorphous morphology	[29]
		8. Phase morphology of polymer blend	[29]
	Barrel	1. Melting behaviour	[32]
	Nozzle	1. Timing of different stages	[28]
		1. Unmelted granules	[22]
	Screw	2. Solid bed/ melt ratio	[22]
		1. Clamping force	[36]
	tie bar	2. Cavity pressure	[27]
Sol-gel Ultrasound Sensors		1. Velocity of polymer melt during solidification	[50]
	Barrel	2. Timing of different process stages	[4,50,53,54]
		3. Polymer degradation	[51]
	&	4. Incomplete filling	[51]
		5. shrinkage	[4]
	Mould-insert	6. Part detachment	[4]
		7. Velocity of flow front	[53]
		8. Mould opening & closing time	[53]
		9. Effects of different process settings on morphology	[54]
		10. Storage modulus	[54]
		1. Screw movements	[56]
		2. Different stages of IM	[52,56]
	Nozzle	3. Static density	[55]
		4. Flow speed	[55]
		5. Effect of feeding stage on the tensile properties of the part	[52]

### 4.2. Overview of Materials for the Fabrication of Ultrasonic Sensors by Sol-Gel Technique

Sol-gel UTs have great potential for industrial IM process monitoring due to both the richness of material and process information that can be extrapolated from the variation in US signal properties and also due to the non-invasive nature of the sensors and the flexibility to apply as a coating in almost any part of the IM machine and tool.

However, a drawback of the sol-gel sensors developed to date is the toxicity and environmentally harmful nature of the materials used. One of most popular materials is lead zirconate titanate (PZT), because of its excellent piezoelectric constant $\left(d_{33}\right)$ 233 ρC/N and a considerable relative dielectric constant of about 1180 [48]. Lead exposure is associated with serious environmental contamination and health problems and finding replacement lead-free materials for use in ultrasound sensing has been a recent area of research. The replacement material should have specific characteristics including high curie temperature to function at high temperatures, and high dielectric constant and piezoelectrical effect to be sensitive as an ultrasonic transducer [57]. In this section, lead-based materials and their performance as ultrasonic sensors is outlined, and then potential lead-free alternatives are discussed.

#### 4.2.1. Lead-Based Sol-Gel Composites

One of the common lead-based composites used in ultrasonic sensors is PZT/PZT, which has a wide range of center frequency from 17 to 160 MHz and a −6 dB bandwidth ranging from 14 to 37.5 MHz. PZT/PZT has been fabricated on various substrates including steel and aluminum [58,59,60]. BiT (Bismuth Titanate) has a high curie temperature of 675 °C and has been applied in a BiT/ PZT composite. BiT/PZT film fabricated on a steel substrate exhibited a signal to noise ratio of 31 dB, and a center frequency of 8 MHz to 13 MHz [53,61]. CaBiT (Calcium–Bismuth–Titanate) ceramic also has a high curie temperature of 788 °C and has also been combined with PZT in fabrication of ultrasound sensors. A 50 μm film composite of CaBiT/PZT was fabricated on titanium substrate as an ultrasonic transducer with a center frequency of 6.3 MHz [62].

Another lead-based sol-gel composite is PMN/PT (Lead Magnesium Niobium–lead titanate), fabricated as a free-standing film with 30 µm thickness and 80MHz centre frequency [63]. A PT/BT film composite (Lead Titanate/Barium Titanate) with a temperature durability of 300 °C was fabricated as an ultrasonic sensor on a titanium substrate with 60 µm thickness and a centre frequency of 32 MHz [64]. The lead-based composite materials used in ultrasonic sensors and their performance are summarized in Table 3.

#### 4.2.2. Lead-Free Sol-Gel Composites

Lead-free sol-gel composites have been developed in an effort to produce more environmentally friendly sensors. Two lead-free materials are the KNN (Potassium–Sodium–Niobate), which has a high curie temperature of 358 °C, and BNT (Bismuth Sodium Titanate) which has high ferroelectrical and polarization properties. The combination of these two lead-free ceramics has been studied by Lau et al. for ultrasonic sensor fabrication [65]. They fabricated a KNN/BNT sensor of 5 µm thickness on a platinum-buffered-Si substrate. This showed good performance as an ultrasonic transducer with 193 MHz center frequency and a −6 dB bandwidth of 34%. Ho Lam et al. [66] developed a KNN/BNT sensor with a higher center frequency of 170 to 320 MHz, and a −6 dB bandwidth of 34% to 64%. The application of BNT alone as an ultrasonic transducer has also been studied [67]; an 11 µm thickness BNT sensor exhibited suitable dielectric and ferroelectric properties and a frequency-bandwidth of 98 MHz.

Three other promising and lead-free materials are CaBiT (Calcium–Bismuth–Titanate) and BiT (Bismuth–Titanate) with high curie temperatures of 788 °C and 675 °C, respectively and BST (Barium–Strontium–Titanate) which has a very high dielectric constant. A BiT/BiT fabricated sensor indicated high thermal durability up to 600 °C, but has a low relative dielectric constant of about 180 [68]. The performance of CBiT/BST and CBiT/BiT ceramic composites as ultrasonic sensors was investigated in [62]. The results showed that CBiT/BST exhibited high dielectric properties although the film quality was poor due to high surface roughness. CBiT/BiT exhibited good sensitivity as a sensor although the poling is challenging due to the low dielectric constant of BiT.

TiO${}_{2}$ with a high dielectric constant has been used to boost the application of BiT as an ultrasonic transducer, and the performance of fabricated BiT/TiO${}_{2}$ on a titanium substrate has been evaluated [69]. The result indicated a temperature durability of about 450 °C and a signal to noise ratio of 20 dB. Another sol-gel material applied with BiT to enhance the properties for application as an ultrasonic transducer is ST (Strontium-Titanate), because of its reasonable dielectric constant and being paraelectric [70]. A 100 µm BiT/ST film presented high-temperature durability of 500 °C and reasonable ultrasonic performance.

LN (Lithium niobate (LiNbO${}_{3}$)) has also been evaluated as a potential material in a LN/BiT composite, due to its considerable curie temperature (1200 °C) [71]. A high operating temperature of 600 °C was shown, and comparing the performance to BiT/BiT, the Ln/BiT performed better as a high-temperature ultrasonic sensor. Lead-free materials investigated for ultrasonic performance are summarized in Table 3. To date, all ultrasound sensors developed for application in the injection moulding process are lead-based, such as PZT/PZT or BiT/PZT; however, these lead-free materials should be investigated as non-toxic alternatives. The highest temperature range in the injection moulding process is from about 180 °C to 300 °C, and all the lead-free materials listed in Table 3 are suitable for this temperature range. The desired ultrasound frequency bandwidth depends on the type of polymer being investigated and can range from 1KHz to 30 MHz. The center frequencies and bandwidths of the materials summarized in Table 3 can be used to select an appropriate sensor material according to the polymers of interest.

## 5. Discussion and Future Directions

The injection moulding industry, like many other manufacturing industries, is undergoing a period of considerable disruption, driven on one hand by an urgent need to enhance sustainability in the use of polymeric materials and on the other hand enabled by progress in sensorization and big data analytics under the framework of Industry 4.0. Traditional petroleum-based polymers are a finite resource and there is increasing pressure to transition to more sustainable raw materials, either through recycling or by the development of novel bio-based polymers with analogous properties [72]. Such materials are more challenging to process, exhibiting either greater variation in properties (in the case of recyclates) or are more sensitive to thermal degradation requiring incorporation of compatibilizers and careful adjustment of processing parameters compared to conventional thermoplastics (in the case of biopolymers) [73,74].

Recently, microcellular injection moulding technologies have gained increasing interest to support the energy-efficient processing of lightweight, foamed parts, which can achieve similar or better mechanical properties while requiring less material than with conventional processing [75]. These material and process developments present increasing process control challenges while producers are also aiming for higher precision products, zero-defect production, reduced set-up and changeover times and faster, more efficient process development.

At the same time, greater sensorization of manufacturing processes combined with Big Data Analytics is recognised as a key pillar of the Industry 4.0 approach, capable of driving improvements in the flexibility and quality of manufacturing processes as well as reducing energy consumption and waste generation [76].

To this end, the application of ultrasound sensors promises to be a key tool in the future of injection moulding processing, as various properties of ultrasound signal propagation can be related to numerous key process and product parameters. As summarised in Table 2, ultrasound transducers can be implemented in many different areas of the injection moulding machine to monitor the operation of all stages of the injection moulding process as well as providing insight into material properties, which influence the quality of the final parts. With the sol-gel fabrication method, ultrasound sensors can be implemented as thin surface coatings, avoiding the challenges of fitting bulky probes into the mould tool. Further, sol-gel transducers can be applied in high temperature areas, such as the nozzle and on the barrel, without the need for a buffer. As outlined in Table 3, recent developments have been made in the identification of alternative lead-free materials suitable for high temperature ultrasound transducers without the issues of high toxicity associated with conventional lead-based probes. These should be explored for future application in the injection moulding process.

While clearly the application of ultrasonic sensors in injection moulding has achieved considerable progress, further studies are needed to meet the demands of more complex materials, processing and part designs. Firstly, fabricated sol-gel ultrasonic sensors are not commercially available, and further study is required to ensure they are robust, reliable and practical to use in industry. Further, process parameters such as pressure and temperature are deduced from ultrasonic sensors indirectly. While the ultrasound signals are very sensitive to variation in material and process parameters it is difficult to measure the absolute values from the ultrasonic propagation properties.

The development of smart calibration techniques to extract these parameters more conveniently is essential to support wider industrial take-up. This will require the incorporation of ultrasound sensors into a wider sensing and modelling framework, ideally in tandem with other novel sensor developments such as the embedding of thermocouple wires in surface coatings using Direct Write Thermal Spray [77].

Recent work demonstrated the potential of multivariate data analysis using multiple in-mould pressure and temperature sensors together with machine process data to predict various product quality indicators using Partial Least Squares (PLS) regression. Good predictions of part weight, thickness and diameter were made under various process perturbations; however, it was noted that the quality changes due to variations in cooling conditions could not be detected using cavity pressure [78]. Ultrasound sensors, however, demonstrate high sensitivity to cooling conditions and could also allow for the prediction of more sophisticated quality indices such as mechanical properties.

Finally, the potential of using this sensor in advanced injection moulding processes such as microcellular injection moulding, which has several advantages over conventional injection moulding for producing more economical lightweight parts with higher mechanical properties, needs further investigation. The optimisation of process parameters to control the foaming process in order to achieve high quality surface finish and mechanical properties is complex, and progress is needed over slow trial-and-error approaches to tuning the process settings. Recent work examined the use of imaging of surface properties post-production to train a Convolutional Neural Network (CNN) to provide recommended process parameter settings in a microcellular injection moulding process to achieve high quality surface condition [79]. However, ultrasound sensors may provide further improvement over such an approach, as characterisation of not only surface finish, but also cell size and skin layer thickness (which affect the dimensional stability and mechanical properties) can be achieved inline rather than post-production.

## 6. Conclusions

This review investigates different types of ultrasonic sensors and their applications in the injection moulding process. The advantages of ultrasonic sensors over conventional temperature and pressure sensors are elaborated. For example, since the pressure drops to zero during cooling, pressure sensors cannot monitor parameters related to the cooling stage, despite this stage being the most crucial in relation to the quality of the product. Temperature sensors, such as thermocouples, are not precise in measurement since they measure the surface temperature rather than the bulk temperature of the melt. Further, ultrasonic sensors have been shown to be sensitive to other physical properties of the material including the degree of the crystallinity in the polymer and the shear and tensile properties of the product.

Conventional ultrasonic probes can monitor various process parameters in real-time (See Table 2), while they have some limitations including being unsuitable for high-temperature areas, requiring modifications in the machine tools, and operating in a limited frequency range. Hence, the ultrasonic sensors fabricated by the sol-gel technique emerged to compensate for these limitations. Different piezoelectric materials (See Table 3) can be employed for the fabrication of these sensors; the more recent exploration of lead-free materials is desired due to the toxicity and environmental damage associated with lead-based materials.

The increasing application of ultrasound sensors in industrial injection moulding processes is envisaged as a promising real-time process and product characterisation tool to drive towards more efficient and sustainable production.

## Figures and Tables

**Table 1:** Summary of Ultrasound propagation properties.

Equation Number	Measured Ultrasonic Properties	Related Material Properties	Reference
(1)–(4)	Longitudinal Ultrasonic Velocity	1. Bulk Moduli	[21,22,23]
		2. Density	
		3. Pressure & Temperature	
(5)–(6)	Velocity of Longitudinal & Shear Waves	1. Density	[24]
		2. Wavelength	
		3. Attenuation	
		4. Ultrasonic Velocity	
(7)–(9)	Reflection & Transmission Coefficient	1. Number of mediums	[25]
		2. Acoustic Impedance	
		3. Density	
		4. Wave velocity	
(10)	Longitudinal Ultrasonic Velocity in solid	1. Young’s Modulus	[19]
		2. Poison’s Ratio	
		3. Material Density	
(11)	Ultrasonic Velocity of two echoes through melt	1. Thickness of Sample	[19]
		2. Echo time	
(12)	Ultrasonic attenuation of two echoes through melt	1. Sample thickness	[19]
		2. Amplitude of signals	
(13)	Ultrasonic Velocity in the tie bar of injection moulding	1. The stress of tie bar	[26,27]
		2. The density of tie bar	
		3. Lamé and Murnaghan constants of the material	

**Table 3:** Lead-free and lead-based sol-gel composites and the ultrasonic properties.

Sol-Gel Composite Material	Center Frequency (MHz)	Temperature-Durability (°C)	−6 dB Bandwidth (%-MHz)	Film Thickness (µm)	Composite Type	Reference
PZT/PZT	17–160	380	16–52%	11–25	Lead-based	[58,59,60]
BiT/PZT	8–13	600	6–8 MHz	-	Lead-based	[53,61]
CaBiT/PZT	6.3	600	-	50	Lead-based	[62]
PMN/PT	82	-	65%	30	Lead-based	[63]
PT/BT	32	300	18 MHz	60	Lead-based	[64]
KNN/BNT	170–320	320	34–64%	6	Lead-free	[65,66]
BNT	98	320	84%	11	Lead-free	[67]
BiT/BiT	-	600	-	50	Lead-free	[68]
CBiT/BiT	6.5	600	-	50	Lead-free	[62]
CBiT/BST	24.9	600	-	50	Lead-free	[62]
BiT/TiO2	10.9	450	4.10%	100	Lead-free	[69]
BiT/ST	-	500	-	100	Lead-free	[70]
LN/BiT	-	700	-	50	Lead-free	[71]

## Data Availability

Not applicable.
